# Exercise Training in Patients with Chronic Respiratory Diseases: Are Cardiovascular Comorbidities and Outcomes Taken into Account?—A Systematic Review

**DOI:** 10.3390/jcm8091458

**Published:** 2019-09-13

**Authors:** Ana Machado, Kirsten Quadflieg, Ana Oliveira, Charly Keytsman, Alda Marques, Dominique Hansen, Chris Burtin

**Affiliations:** 1 REVAL—Rehabilitation Research Center, Faculty of Rehabilitation Sciences, Hasselt University, 3590 Diepenbeek, Belgium; 2 Respiratory Research and Rehabilitation Laboratory (Lab3R), School of Health Sciences (ESSUA), University of Aveiro, 3810 Aveiro, Portugal; 3 Respiratory Medicine, West Park Healthcare Centre, Toronto, ON M6M 2J5, Canada; 4 School of Rehabilitation Sciences, Faculty of Health Sciences, McMaster University, Hamilton, ON L8S 4K1, Canada; 5 BIOMED—Biomedical Research Institute, Hasselt University, 3590 Diepenbeek, Belgium; 6 Institute of Biomedicine (iBiMED), University of Aveiro, 3810 Aveiro, Portugal; 7 Jessa hospital, Heart Centre Hasselt, 3500 Hasselt, Belgium

**Keywords:** exercise, chronic lung disease, chronic obstructive pulmonary disease, COPD, asthma, interstitial lung disease, ILD, cardiovascular comorbidities, cardiovascular outcomes

## Abstract

Patients with chronic obstructive pulmonary disease (COPD), asthma and interstitial lung diseases (ILD) frequently suffer from cardiovascular comorbidities (CVC). Exercise training is a cornerstone intervention for the management of these conditions, however recommendations on tailoring programmes to patients suffering from respiratory diseases and CVC are scarce. This systematic review aimed to identify the eligibility criteria used to select patients with COPD, asthma or ILD and CVC to exercise programmes; assess the impact of exercise on cardiovascular outcomes; and identify how exercise programmes were tailored to CVC. PubMed, Scopus, Web of Science and Cochrane were searched. Three reviewers extracted the data and two reviewers independently assessed the quality of studies with the Quality Assessment Tool for Quantitative Studies. MetaXL 5.3 was used to calculate the individual and pooled effect sizes (ES). Most studies (58.9%) excluded patients with both stable and unstable CVC. In total, 26/42 studies reported cardiovascular outcomes. Resting heart rate was the most reported outcome measure (*n* = 13) and a small statistically significant effect (*ES* = −0.23) of exercise training on resting heart rate of patients with COPD was found. No specific adjustments to exercise prescription were described. Few studies have included patients with CVC. There was a lack of tailoring of exercise programmes and limited effects were found. Future studies should explore the effect of tailored exercise programmes on relevant outcome measures in respiratory patients with CVC.

## 1. Background

Chronic obstructive pulmonary disease (COPD), asthma and interstitial lung diseases (ILD) are among the most representative chronic respiratory diseases in the world [1,2]. These diseases affect over 1 billion people and have a significant impact on patients’ disability and quality of life (9.5% of the disability-adjusted life years in 2010 [3]), being a leading contributor to disease burden and one of the top causes of death worldwide (over 3 million deaths in 2016) [2,3,4].

In recent years, the association between chronic respiratory diseases and cardiovascular diseases has attracted huge interest in clinical research [5]. Cardiovascular diseases (e.g., arterial hypertension, coronary artery disease, congestive heart failure, peripheral vascular disease and pulmonary hypertension) are among the most prevalent and impactful comorbidities in patients with COPD (13%–68% of population), asthma (3%–25% of population) and ILD (8%–86% of population) [6,7,8,9,10,11,12,13]. Namely, they are responsible for further impairing patients’ functional status and health-related quality of life, increasing the risk of hospitalization and mortality (hazard ratio 1.1–3.4 [14,15,16,17]), and contributing to a higher economic and societal burden, and worse prognosis [7,9,10,12]. The need to look beyond the lungs while treating these patients is, therefore, evident [18]. In this regard, a comprehensive assessment and management of these cardiovascular comorbidities with tailored interventions has been recommended [7,12,19,20].

Exercise training is a cornerstone intervention in both pulmonary and cardiac rehabilitation [21,22]. It relieves symptoms and improves functionality, exercise tolerance and health-related quality of life in patients with chronic respiratory and cardiovascular diseases [21,22], and therefore might be a promising intervention for the management of patients with these co-occurring conditions. Nevertheless, studies have shown that these effects are usually reduced in patients suffering from chronic respiratory diseases with accompanying cardiovascular comorbidities compared to those without cardiovascular comorbidities [7,19,23]. Moreover, recommendations on how to adjust exercise programmes to co-existing cardiovascular conditions in COPD, asthma and ILD are scarce. 

Therefore, in order to inform evidence-based statements, this systematic review aimed to: (i) identify the eligibility criteria in terms of cardiovascular disease that have been used to refer patients with COPD, asthma and ILD for studies investigating the effectiveness of exercise programmes of at least 3 months; (ii) assess the impact of at least 3 months of exercise training on cardiovascular outcomes in these patients; and (iii) identify how the exercise programmes have been tailored to patients’ cardiovascular comorbidities.

## 2. Methods

### 2.1. Search Strategy

This systematic review was reported according to the Preferred Reporting Items for Systematic reviews and Meta-analyses (PRISMA) guidelines [24] and was conducted in two phases. Phase 1 identified the eligibility criteria that have been used to select patients with cardiovascular comorbidities in clinical trials investigating the effectiveness of exercise programmes. Phase 2 assessed the impact of exercise training on cardiovascular outcomes, and identified how the exercise programmes have been tailored to patients’ cardiovascular comorbidities.

A systematic literature search was performed in May 2019 on the following electronic databases: PubMed, Scopus, Web of Science and Cochrane. The search terms were limited to titles, abstracts and keywords/MeSH terms. The full search strategy is presented in Appendix A. 

### 2.2. Eligibility Criteria and Study Selection

For phase 1, studies were included if they (i) studied adult patients with stable COPD, asthma and/or ILD (i.e., 4 weeks without exacerbations); (ii) implemented at least 12 weeks of exercise training (i.e., endurance and/or strength training) as an intervention [25]; (iii) implemented at least 2 directly supervised exercise sessions per week [26]; (iv) were original prospective quantitative studies; and (v) were written in Portuguese, English, French, Dutch or Spanish languages. Retrospective studies, case studies, case series, abstracts and studies involving alternative modalities of exercise (e.g., yoga, tai chi, qigong) were excluded. After removing duplicates, three reviewers (AM, KQ and AO) assessed all the potential studies identified. Studies were selected based on their titles and abstracts. When the title and abstract were potentially relevant to the purpose of the review, the full text was read carefully to decide on its inclusion. A fourth reviewer (CB) was consulted to solve any disagreements.

For phase 2, studies included in phase 1 that specified the prevalence of cardiovascular comorbidities (i.e., any cardiovascular condition co-existing with the respiratory disease, identified by doing an objective patients’ assessment, checking their medical records or ask patients to self-report their comorbidities) in the baseline characteristics of the population under study and/or reported at least one cardiovascular outcome (i.e., heart rate, systolic and diastolic blood pressure, flow-mediated dilation, pulse-wave velocity, intima thickness of arteria carotid, cardiac function and structure, heart rate variability, ECG analysis and blood lipid profile) were included. 

### 2.3. Quality Assessment and Data Extraction

Two reviewers (KQ and AO) independently assessed the quality of the studies included in phase 2 with the Quality Assessment Tool for Quantitative Studies, developed by the Effective Public Health Practice Project, Canada [27]. This tool assesses six domains of methodological quality: (i) selection bias; (ii) study design; (iii) confounders; (iv) blinding; (v) data collection methods; and (vi) withdrawals and dropouts [27]. Each domain is rated as “strong”, “moderate” or “weak”, according to a standardized guide, and the overall rating of the study is determined based on the total number of “strong” and “weak” scores [27].

In phase 1, data regarding the eligibility criteria (i.e., inclusion and exclusion criteria) used to select patients for the study were extracted from all included studies. Afterwards, all conditions that would preclude patients’ participation in the exercise programmes, reported either as reasons for inclusion (e.g., absence of severe cardiovascular disease) or exclusion (e.g., presence of severe cardiovascular disease) of these patients, were compiled and reported as exclusion criteria. Additionally, data from the studies included in phase 2 were extracted in a predesigned structured table format comprising the following topics: study (first author, year of publication, country); study design; population (number of participants, diagnosis, age, gender, forced expiratory volume in 1 s (FEV_1_), forced vital capacity (FVC), diffusing capacity for carbon monoxide (DLCO)); intervention (type and intensity of intervention); duration and frequency (duration of the intervention, duration and frequency of sessions); outcome and outcome measure; and results. For the scope of this review, only cardiovascular outcomes and outcome measures were considered.

### 2.4. Data Analysis and Synthesis

Inter-rater agreement analysis using Cohen’s kappa was used to explore the consistency of the quality assessment performed by the two reviewers. The value of Cohen’s kappa ranges from 0 to 1 and can be interpreted as slight (≤0.2), fair (0.21–0.4), moderate (0.41–0.6), substantial (0.61–0.8), or almost perfect (≥0.81) agreement [28]. The statistical analysis was performed using IBM SPSS 24.0 (IBM, Armonk, New York, NY, USA).

Whenever possible, effect sizes (ES) were calculated and a meta-analysis was performed. ES were calculated as Cohens’ d based on the Pre/Post means and standard deviations or mean differences and standard deviations, according to the formula of Morris [29], and interpreted as small (≥0.2), medium (≥0.5) or large (≥0.8) [30]. Meta-analysis was performed on MetaXL 5.3. Pooled effect estimates were calculated with the inverse variance technique assuming a fixed-effects model. The input data were the Cohen’s d value of each study and the respective standard error. The output was the pooled Cohen’s d value and corresponding confidence intervals. Homogeneity among the studies was evaluated using Cochran’s Q test and the *I*^2^ statistic.

## 3. Results

### 3.1. Study Selection

**Figure 1:**
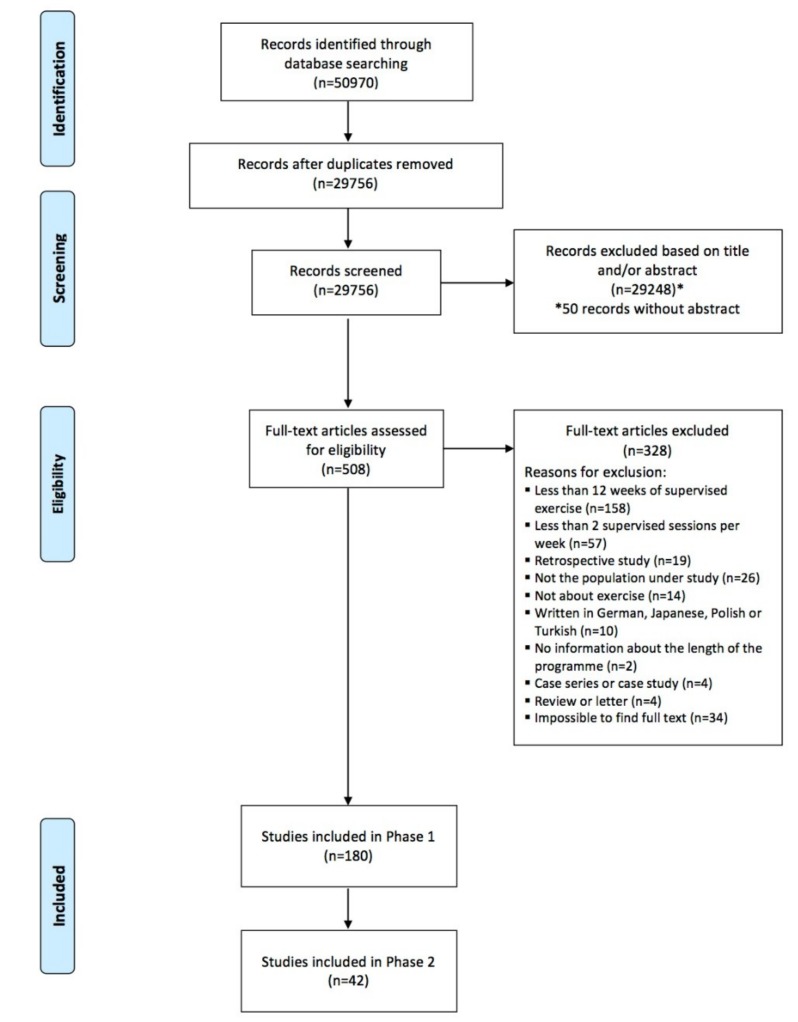
PRISMA flowchart of the included studies.

The literature search provided a total of 50.970 records. After duplicates removal, 29.756 records were screened for relevant content through title and abstract and 29.248 were excluded. The full text of 508 potentially relevant articles was assessed. From these, 180 articles were included in phase 1 and 42 in phase 2 (Figure 1).

### 3.2. Phase 1: Criteria Used to Exclude Patients with Cardiovascular Comorbidities from Exercise Programmes

The 180 studies included were conducted between 1987 and 2019. In total, 156 studies included patients with COPD [23,31,32,33,34,35,36,37,38,39,40,41,42,43,44,45,46,47,48,49,50,51,52,53,54,55,56,57,58,59,60,61,62,63,64,65,66,67,68,69,70,71,72,73,74,75,76,77,78,79,80,81,82,83,84,85,86,87,88,89,90,91,92,93,94,95,96,97,98,99,100,101,102,103,104,105,106,107,108,109,110,111,112,113,114,115,116,117,118,119,120,121,122,123,124,125,126,127,128,129,130,131,132,133,134,135,136,137,138,139,140,141,142,143,144,145,146,147,148,149,150,151,152,153,154,155,156,157,158,159,160,161,162,163,164,165,166,167,168,169,170,171,172,173,174,175,176,177,178,179,180,181,182,183,184,185], 15 studies included patients with asthma [46,52,63,78,185,186,187,188,189,190,191,192,193,194,195] and 16 studies included patients with ILD [87,185,196,197,198,199,200,201,202,203,204,205,206,207,208,209].

**Figure 2:**
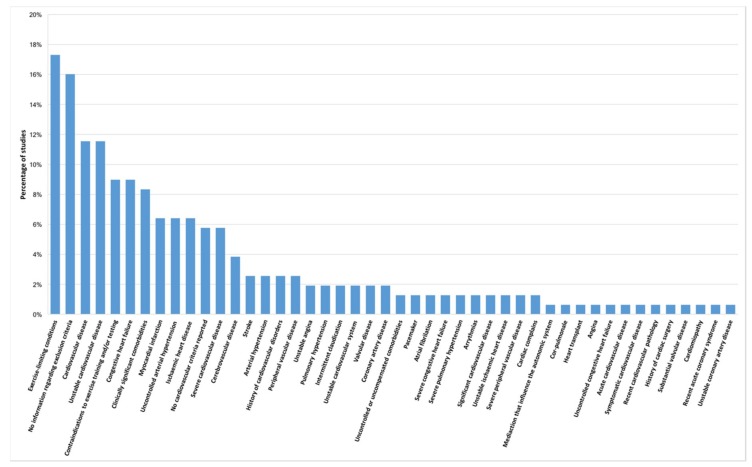
Exclusion criteria reported in studies with patients with chronic obstructive pulmonary disease (COPD) (*n* = 156 studies).

Forty-four different exclusion criteria were found in studies with patients with COPD (Figure 2). From these, exercise-limiting conditions [34,36,40,53,60,61,89,92,104,114,125,134,135,138,148,157,166,167,168,169,170,173,174,179,181,183,184] (*n* = 27; 17.3%) was the most reported exclusion criterion, followed by general cardiovascular disease [32,39,44,70,74,75,94,100,103,107,109,112,130,152,153,160,164,171] (*n* = 18; 11.5%) and unstable cardiovascular disease [38,47,53,65,66,98,99,101,108,116,117,118,119,133,137,146,147,180] (*n* = 18; 11.5%). Thirty-four (21.8%) of the studies [23,48,50,55,63,67,69,73,77,78,79,80,83,84,86,88,91,95,97,110,111,115,122,126,127,132,142,149,150,155,161,165,175,185] did not report any information regarding the eligibility criteria for cardiovascular comorbidities.

**Figure 3:**
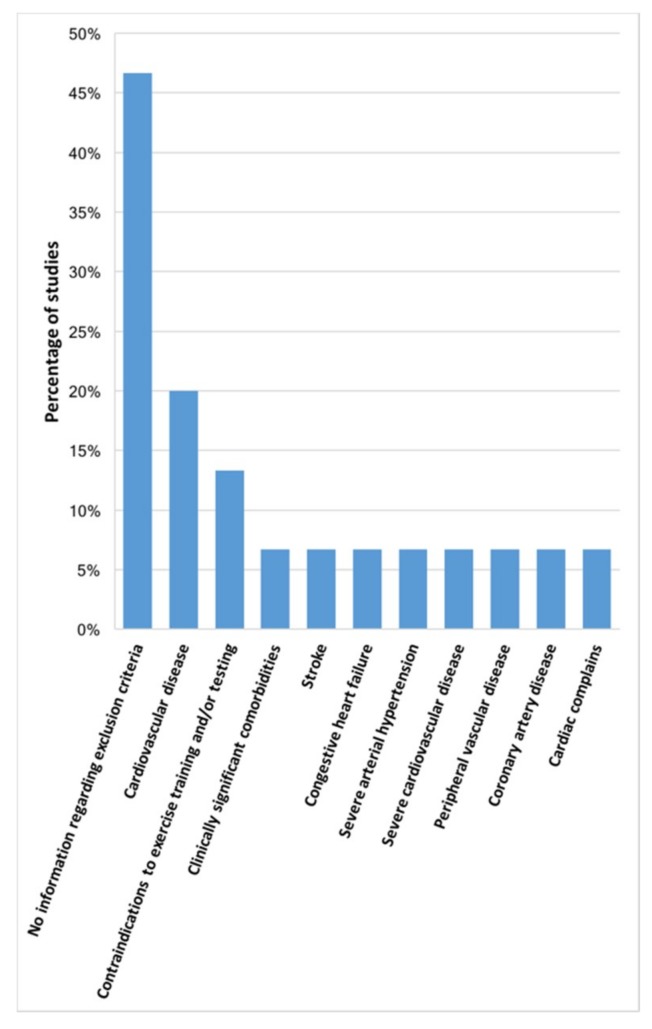
Exclusion criteria reported in studies with patients with asthma (*n* = 15 studies).

In studies including patients with asthma, 10 different exclusion criteria were found (Figure 3). General cardiovascular disease [186,191,192] (*n* = 3; 20.0%) was the most reported criterion, followed by contraindications to exercise training and/or testing [194,195] (*n* = 2; 13.3%). Seven (46.7%) studies [63,78,185,187,189,190,193] did not report information about exclusion criteria.

**Figure 4:**
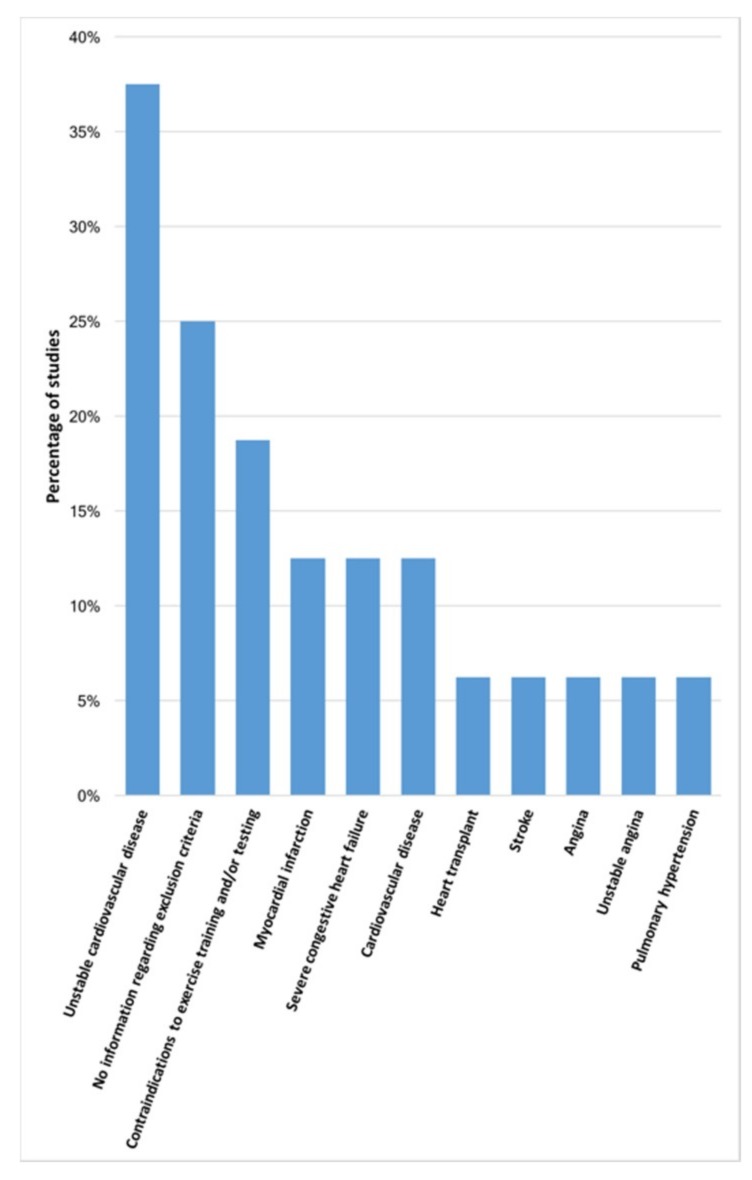
Exclusion criteria reported in studies with patients with interstitial lung diseases (ILD) (*n* = 16 studies).

Studies in patients with ILD reported 10 different exclusion criteria (Figure 4). From these, unstable cardiovascular disease [198,199,202,203,208,209] (*n* = 6; 37.5%) was the most reported criterion, followed by contraindications to exercise training and/or testing (e.g., unstable angina, recent myocardial infarction or cerebrovascular accident) [201,204,207] (*n* = 3; 18.8%). Four (25.0%) studies [185,197,205,206] did not report any information about exclusion criteria.

In general, 22.8% of the studies [23,48,50,55,63,67,69,73,77,78,79,80,83,84,86,88,91,95,97,110,111,115,122,126,127,132,142,149,150,155,161,165,175,185,187,189,190,193,197,205,206] did not report information about the eligibility criteria, 18.3% of the studies [33,42,54,65,66,72,82,96,98,99,101,108,116,117,118,119,128,137,141,143,145,146,180,182,194,195,200,201,202,203,207,208,209] only excluded patients with acute or unstable cardiovascular comorbidities that contraindicated exercise training, and 58.9% of the studies [31,32,34,35,36,37,38,39,40,41,43,44,45,46,47,49,51,52,53,56,57,58,59,60,61,62,64,68,70,71,74,75,76,81,85,87,89,90,92,93,94,100,102,103,104,105,106,107,109,112,113,114,120,121,123,124,125,129,130,131,133,134,135,136,138,139,140,144,147,148,151,152,153,154,156,157,158,159,160,162,163,164,166,167,168,169,170,171,172,173,174,176,177,178,179,181,183,184,186,188,191,192,196,198,199,204] excluded both stable and unstable cardiovascular comorbidities.

### 3.3. Phase 2: Impact of Exercise Training on Cardiovascular Outcomes and Design of the Exercise Programmes

#### 3.3.1. Quality Assessment

Results of the methodological quality assessment are presented in Table 1. Most of the studies (*n* = 24; 57.1%) were of weak quality. The agreement between the two reviewers was substantial (k = 0.72; 95%CI = 0.53–0.91; *p* < 0.001; percentage of agreement = 85.7%).

#### 3.3.2. Study Characteristics

Characteristics of the included studies are shown in Table 2, Table 3 and Table 4. From the 42 included studies, 32 studies included patients with COPD [33,37,42,43,44,48,49,53,54,55,57,59,68,80,85,90,101,104,106,107,108,120,121,123,124,128,129,136,146,147,151,167], two studies included patients with asthma [186,189] and eight studies included patients with ILD [196,198,201,202,203,207,208,209]. Most studies were randomized controlled trials (*n* = 24; 57.1%) [37,43,44,49,53,80,85,120,121,123,124,129,146,147,151,167,186,189,196,198,202,207,208,209].

In total, 1704 patients (65.2% male; data gathered from 34 studies) with a weighted mean age of 65.4 years old and a mean FEV_1_ of 53.7% of predicted (data gathered from 36 studies) were enrolled in the included studies.

Only 13 studies in patients with COPD [33,42,43,44,48,54,55,59,68,85,101,151,167] and six studies in patients with ILD [196,198,203,207,208,209] specified the presence of patients with cardiovascular comorbidities on the population’s baseline characteristics. No studies reporting to include patients with cardiovascular comorbidities were found in asthma. Studies in patients with COPD included patients suffering from arterial hypertension [33,42,43,44,48,54,68,85,151] (nine studies; 314 patients), cardiovascular diseases [43,44,48,55,59,85,101,151,167] (nine studies; 247 patients), circulatory problems [42,43,44,85] (four studies; 84 patients), coronary heart disease [42] (one study; 54 patients), congestive heart failure [33,54] (two studies; 10 patients), dyslipidaemia [33,54] (two studies; 11 patients) and ischemic cardiomyopathy [33] (one study; three patients). Studies in patients with ILD included patients suffering from arterial hypertension [196,203,207,208,209] (five studies; 49 patients), coronary heart disease [207,208,209] (three studies; 21 patients), congestive heart failure [203] (one study; two patients), pulmonary hypertension [207,208,209] (three studies; 15 patients) and history of heart disease [198] (one study; one patient). From these, only three studies [196,207,209] conducted in patients with ILD reported cardiovascular outcomes and outcome measures.

Nineteen studies in patients with COPD [37,49,53,57,80,90,104,106,107,108,120,121,123,124,128,129,136,146,147], two studies in asthma [186,189] and five studies in ILD [196,201,202,207,209] reported cardiovascular outcomes and outcome measures. Studies conducted in patients with COPD presented a large variety of outcome measures, while studies conducted in patients with asthma were mainly focused on blood lipid profile [187,190] (*n* = 2) and studies in patients with ILD reported mainly resting heart rate [196,201,209] (*n* = 3) and blood pressure [196,207,209] (*n* = 3). The most reported outcome measure was resting heart rate [37,57,90,106,107,121,123,124,129,136,196,201,209] (*n* = 13; *ES* = [−0.63; 0.11]). 

**Figure 5:**
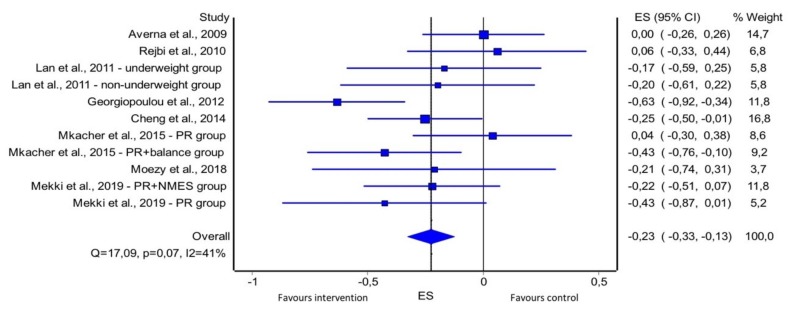
Forest plot of comparison control versus intervention in patients with COPD; outcome: resting heart rate. ES, effect size; NMES, neuromuscular electrical stimulation; PR, pulmonary rehabilitation.

Most studies (*n* = 20; 71.4%) presented only small to moderate effects in the cardiovascular outcome measures reported. Standard deviation of RR intervals [49,108] (*n* = 2; *ES* = [0.67; 2.64]) and root mean square of successive RR interval differences [49,57,108] (*n* = 3; *ES* = [0.69; 2.64]) were the outcome measures presenting the larger effects. In patients with COPD, the effects of exercise training programmes on resting heart rate resulted in an overall pooled *ES* of −0.23 (95% confidence interval −0.33 to −0.13) (Figure 5).

Regarding the exercise programmes, most studies conducted in patients with COPD performed a pulmonary rehabilitation programme [33,54,55,57,59,68,80,90,101,104,106,107,121,123,136,167] (*n* = 16) or an exercise programme combining aerobic and strength training [38,43,44,45,54,86,129,130] (*n* = 8). Sessions were conducted 2–6 times per week and each session lasted from 15 min to 2 h. Programme duration varied between 12 weeks and 18 months. A wide range of intensities was used to prescribe the exercise: 60%–80% of the maximum heart rate, 50%–100% of the peak or maximum oxygen uptake, 50%–125% of the peak or maximum workload, 35%–75% of one-repetition maximum, dyspnoea and perceived exertion levels between 3–6 on the modified Borg scale and 12–16 on the Borg scale. None of the studies specified any adjustments to tailor the exercise programmes to patients’ cardiovascular comorbidities. Only one study [108] described adjusting the training programme in different mesocycles in order to improve specific cardiovascular outcomes.

Studies conducted in patients with asthma performed either an exercise programme combining aerobic and strength training for 3 months [189] or aerobic training for 6 months [186]. Sessions occurred 3 times/week, for 30 min each, at an intensity of 60%–80% of the maximum heart rate. No specific adjustments to improve specific cardiovascular outcomes were reported.

In patients with ILD, most studies conducted exercise programmes combining aerobic and strength training [201,202,203,207,208,209] (*n* = 6). The majority of programmes lasted for 12 weeks with 2 sessions/week [196,198,202,203,207,208,209] (*n* = 7). Sessions had a duration of 60–90 min and exercise was prescribed at an intensity of 65%–85% of maximum heart rate, 50%–90% of peak workload, dyspnoea and perceived exertion levels between 3 and 6 on the modified Borg scale. None of the studies specified any adjustments to tailor the exercise programmes to patients’ cardiovascular comorbidities. 

## 4. Discussion

To the best of the authors’ knowledge, this is the first comprehensive overview of the scientific literature summarizing (i) the eligibility criteria in terms of cardiovascular disease used to select patients with chronic respiratory disease to exercise training studies, (ii) the impact of at least 3 months of exercise training on cardiovascular outcomes, and (iii) adjustments made to tailor exercise training prescription to patients with cardiovascular comorbidities. It was found that (i) in the majority of the studies (58.9%) patients with cardiovascular comorbidities were excluded a priori, (ii) there is limited evidence about the impact of exercise training on cardiovascular outcomes in patients with chronic respiratory diseases, and (iii) none of the studies explicitly mentioned how to tailor exercise training modalities in light of cardiovascular comorbidities.

A large diversity was found regarding the cardiovascular conditions that are used as exclusion criteria in exercise-related research. Interestingly, the majority of the exclusion criteria reported (34/45) are not considered contraindications to exercise training. Indeed, just a minority of the studies (18.3%) excluded only patients with acute/unstable cardiovascular disease that contraindicated participation in exercise training. Most studies excluded patients with both stable and unstable cardiovascular comorbidities, although at least 20%–50% of the patients with COPD, asthma or ILD present cardiovascular comorbidities [5,7,18,20,210,211]. Thus, by excluding patients with cardiovascular comorbidities or any other comorbidity that does not present any contraindication to perform exercise training, translation of knowledge to clinical practice can only be done for a subset, or sometimes even a minority, of patients. This finding might have a far-reaching consequence, namely that current knowledge (including clinical guidelines) is disease-centred and, thus, inadequate to sufficiently support/guide clinicians on how to prescribe exercise for patients with chronic respiratory diseases and multiple chronic conditions [13,212]. Furthermore, some of the criteria reported (e.g., cardiovascular disease) were too vague to allow understanding of which conditions were really excluded and over 20% of the included studies did not report any information concerning to eligibility criteria, even though this is key information to ensure clarity and transparency of the research [213].

Exercise training programmes in patients with cardiovascular comorbidities resulted in significant improvements in general reported outcomes, namely symptoms, functionality, exercise capacity, muscle strength and health-related quality of life, comparable to the ones usually found in respiratory patients [22]. However, regarding cardiovascular outcomes, in the majority of the studies (71.4%) only small to moderate effects were found, with the larger effects being reported for heart rate variability measurements (*ES* = [−0.78; 2.64]) and blood lipid profile (*ES* = [−2.31; 0.62]). Additionally, a small but significant overall effect of exercise training programmes on resting heart rate of patients with COPD was found. These results are yet not inferior to the ones previously reported for patients with cardiovascular diseases, in whom beneficial effects of exercise training have been found for heart rate variability and heart rate recovery [214], and inconsistent but significant and modest effects have been reported for arterial blood pressure and blood lipid profile [214,215,216,217]. We hypothesized that several reasons might be contributing to the limited effects found. First, most studies have not reported any specific adjustments in the exercise prescription to tailor the programme to patients’ cardiovascular comorbidities, although it is plausible that they have made some adjustments without specific reporting in the published paper. It is known that cardiovascular conditions require specific considerations when formulating the exercise plan [22], and different recommendations exist based on the prevalent cardiovascular disease (e.g., coronary artery disease, congestive heart failure, peripheral arterial disease, pulmonary arterial hypertension) and its severity [218]. Indeed, it is mandatory to tailor exercise duration, frequency, mode, intensity and monitoring to patients’ specificities and needs, clinical conditions, cardiovascular phenotype (risk factors and diseases), fitness level, medication intake (beta blockers, statins, glinides, sulfonylurea), abnormal responses to exercise (myocardial ischemia, atrial fibrillation, ventricular tachycardia) and rehabilitation goals [1,218,219,220,221]. Moreover, the impact of exercise training relies on this proper tailoring of the exercise programme, since it has been shown that different exercise prescriptions result in significant differences in clinical outcomes [221]. Future studies should therefore assess the impact of exercise programmes specifically tailored to patients with co-occurring respiratory disease and cardiovascular comorbidities [7] and report the intervention in detail. Second, guidelines for cardiac rehabilitation from the leading scientific societies recommend that exercise should progress from moderate to vigorous intensity, three times per week [21]. Nevertheless, in some of the included studies, patients exercised at lower intensities and/or fewer times per week, which might have also contributed to the relative lack of effects since the minimum dose of exercise for cardiovascular benefits (>150 min/week of endurance training, energy expenditure 1000–2000 kcal/week) might have not been reached [218,222]. Third, some of the included studies only used strength training in their exercise programmes. Indeed, strength training has been recommended in patients with cardiovascular diseases, but as an adjunct to aerobic training, the last being a core component in these patients’ rehabilitation [21]. From these observations, it became clear that current exercise prescription to patients with COPD, asthma or ILD with cardiovascular comorbidities is far from optimal and deserves significant reconsideration. Nonetheless, digital support on how to prescribe exercise in these patients in accordance to all the different clinical guidelines for different cardiovascular diseases is available, and thus could be used to support health professionals [218]. Lastly, most studies including patients with cardiovascular comorbidities only focused on the assessment of resting heart rate. Although this is a relevant outcome measure and results from meta-analysis in patients with COPD favour intervention, recommendations for patients with cardiovascular diseases advocate a more comprehensive assessment, including outcomes such as arterial blood pressure, blood lipid profile or echocardiography, that are also more in line with the aims of rehabilitation in these patients [223,224]. Therefore, outcomes should be better targeted to patients’ cardiovascular comorbidities [19].

Besides the known prevalence and increased risk of morbidity and mortality that cardiovascular comorbidities impose on patients with chronic respiratory diseases [7,211], only three studies [196,207,209] (all conducted in the last 5 years) included patients with cardiovascular comorbidities and assessed cardiovascular outcome measures. This denotes the current gap in the literature regarding exercise programmes and emphasises the need for specific studies focusing on cardiovascular outcomes in these patients.

This systematic review has a number of limitations that need to be acknowledged. First, as it was anticipated that a large number of studies would be found, only exercise programmes lasting at least 12 weeks were included, which might have led to the loss of other relevant studies. Nevertheless, 12 weeks has been recommended as the minimum exercise duration required to reach benefits in patients with cardiovascular disease [25]. Second, as only few studies including patients with ILD were found, all types of ILD were grouped, although different types of ILD present different characteristics and possibly different cardiovascular comorbidities and responses to exercise training programmes. Third, most of the included studies were of weak quality. Nonetheless, since in exercise interventions blinding of participants is impossible and patients are usually referred by physicians to ensure their safety, it was virtually impossible to ensure strong quality in the quality assessment tool used.

## 5. Conclusions

Although a large number of studies explored the effects of at least 3 months of exercise training in patients with chronic respiratory diseases, only few included patients with cardiovascular comorbidities. Limited effects of the exercise programmes were found on cardiovascular outcome measures, possibly due to the lack of tailoring of the exercise training prescription and comprehensiveness of the cardiovascular outcome measures. Future studies focusing on patients with combined respiratory and cardiovascular diseases and exploring the effects of exercise programmes specifically tailored to these patients are needed to bridge the gap in the literature.

## Figures and Tables

**Table 1:** Quality assessment of the studies included in phase 2 (*n* = 42) with the Quality Assessment Tool for Quantitative Studies.

Study	Selection Bias	Study Design	Confounders	Blinding	Data Collection Method	Withdrawals and Drop-Outs	Global Rating
Cochrane et al., 1990	3	1	1	3	1	3	3
Berry et al., 1999	2	2	3	3	1	1	3
Foy et al., 2001	3	1	3	3	1	1	3
Berry et al., 2003	2	1	1	2	1	1	1
Panton et al., 2004	2	1	3	3	1	1	3
Marquis et al., 2008	2	1	3	1	1	3	3
Averna et al., 2009	3	1	1	3	1	1	2
Berry et al., 2010	3	1	1	2	1	1	2
Rejbi et al., 2010	2	1	2	3	1	1	2
Camillo et al., 2011	2	1	1	3	1	1	2
Lan et al., 2011	3	2	2	3	1	3	3
Corhay et al., 2012	3	2	3	2	1	2	3
Georgiopoulou et al., 2012	2	2	NA	3	1	1	2
Lan et al., 2013	2	2	NA	3	1	3	3
Cheng et al., 2014	3	2	NA	3	1	3	3
Gaunaurd et al., 2014	3	1	1	3	1	1	3
Vainshelboim et al., 2014	3	1	1	3	1	1	3
Borghi-Silva et al., 2015	2	1	1	2	1	1	1
Campos et al., 2015	2	2	NA	3	1	1	2
Leite et al., 2015	2	1	1	3	1	3	3
Marcellis et al., 2015	2	2	NA	3	1	2	2
Mkacher et al., 2015	2	1	1	3	1	1	2
Spielmanns et al., 2015	3	1	1	2	1	3	3
Vainshelboim et al., 2015	2	1	1	3	1	1	2
Boström et al., 2016	3	1	1	2	1	1	2
Cardoso et al., 2016	2	1	3	3	1	1	3
El-Kader et al., 2016	3	1	1	3	1	1	3
Engel et al., 2016	3	1	1	1	1	1	2
Boeselt et al., 2017	3	1	1	3	1	2	3
Kanao et al., 2017	3	2	NA	3	1	1	3
Pacheco et al., 2017	2	3	NA	3	1	1	3
Papp et al., 2017	3	1	3	3	1	2	3
Vainshelboim et al., 2017	2	1	1	3	1	1	2
Vasilopoulou et al., 2017	3	1	3	3	1	1	3
Lan et al., 2018	3	2	NA	3	1	1	3
Moezy et al., 2018	3	1	1	3	1	1	3
Naz et al., 2018a	2	2	NA	3	1	1	2
Naz et al., 2018b	3	1	1	3	1	1	3
Silva et al., 2018	2	1	1	3	1	1	2
Charikiopoulou et al., 2019	2	2	3	3	1	1	3
Mekki et al., 2019	2	1	1	2	1	2	2
Silva et al., 2019	2	1	1	3	1	1	2

Legend: 1 = strong quality; 2 = moderate quality; 3 = weak quality; NA, not applicable.

**Table 2:** Characteristics of the studies in patients with COPD included in phase 2 (i.e., studies that specified the prevalence of cardiovascular comorbidities in the baseline characteristics of the population under study and/or reported at least one cardiovascular outcome) (*n* = 32).

Study and Country	Study Design	Population	Intervention	Duration and Frequency	Cardiovascular Outcomes and Outcome Measures	Results on Cardiovascular Outcomes
Berry et al., 1999 United States of America	Non-controlled study	151 patients with COPD Mild disease group: 99 (54♂; 67.4 ± 6.1 years; FEV_1_ 68.0 ± 1.2%pred) Arterial hypertension: *n* = 44 Circulatory problems: *n* = 14 Coronary heart disease: *n* = 34 Moderate disease group: 36 (22♂; 68.3 ± 6.2 years; FEV_1_ 41.9 ± 0.7%pred) Arterial hypertension: *n* = 16 Circulatory problems: *n* = 5 Coronary heart disease: *n* = 12 Severe disease group: 16 (10♂; 66.1 ± 5.6 years; FEV_1_ 30.1 ± 0.9%pred) Arterial hypertension: *n* = 7 Circulatory problems: *n* = 2 Coronary heart disease: *n* = 8	All groups: Aerobic and strength training Dyspnoea 3–4 in the mBorg	All groups: 12 weeks 3 sessions/week 1 h/session		
Foy et al., 2001 United States of America	Randomized controlled trial	140 patients with COPD Short-term intervention group: 70 (39♂; 66.9 ± 5.9 years; FEV_1_ 59.1 ± 17.2%pred) Arterial hypertension: *n* = 29 Circulatory problems: *n* = 14 Cardiovascular disease: *n* = 27 Long-term intervention group: 70 (39♂; 68.4 ± 6.0 years; FEV_1_ 57.6 ± 18.4%pred) Arterial hypertension: *n* = 32 Circulatory problems: *n* = 9 Cardiovascular disease: *n* = 24	All groups: Aerobic and strength training Dyspnoea 3–4 in the mBorg	3 sessions/week 55–65 min/session Short-term intervention group: 12 weeks Long-term intervention group: 72 weeks		
Berry et al., 2003 United States of America	Randomized controlled trial	140 patients with COPD Short-term intervention group: 70 (39♂; 66.9, 95%CI (65.5; 68.3) years; FEV_1_ 59.1, 95%CI (55.0; 63.2)%pred) Arterial hypertension: *n* = 29 Circulatory problems: *n* = 14 Cardiovascular disease: *n* = 27 Long-term intervention group: 70 (39♂; 68.4, 95%CI (67.0; 69.8) years; FEV_1_ 57.6, 95%CI (53.2; 62.0)%pred) Arterial hypertension: *n* = 32 Circulatory problems: *n* = 9 Cardiovascular disease: *n* = 24	All groups: Aerobic and strength training Dyspnoea 3–4 in the mBorg	3 sessions/week 1 h/session Short-term intervention group 1: 3 months Long-term intervention group: 18 months		
Panton et al., 2004 United States of America	Non-randomized controlled trial	17 patients with COPD Aerobic training group: 8 (2♂; 63.0 ± 8.0 years; FEV_1_ 39.5 ± 31.9%pred) Aerobic+strength training group: 9 (6♂; 61.0 ± 7.0 years; FEV_1_ 41.9 ± 16.0%pred)	Aerobic training group: Aerobic training 50%–70% of HR reserve Aerobic+strength training group: Aerobic and strength training 50%–70% of HR reserve	12 weeks Aerobic training group: 2 sessions/week 60 min/session Aerobic + strength training group: 4 sessions/week (2 of each training) 45–60 min/session	Rate pressure product	Aerobic training group: Pre 177.0 ± 29.0 vs. Post 186.0 ± 30.0, *p* > 0.05 *ES* = 0.31 Aerobic+strength training group: Pre 195.0 ± 35.0 vs. Post 199.0 ± 35.0, *p* > 0.05 *ES* = 0.11
					Total blood cholesterol (mg/dl)	Aerobic training group: Pre 217.0 ± 46.0 vs. Post 217.0 ± 46.0, *p* > 0.05 *ES* = 0.00 Aerobic+strength training group: Pre 201.0 ± 34.0 vs. Post 193.0 ± 23.0, *p* > 0.05 *ES* = −0.28
					Cholesterol – HDL (mg/dl)	Aerobic training group: Pre 62.0 ± 21.0 vs. Post 62.0 ± 20.0, *p* > 0.05 *ES* = 0.00 Aerobic+strength training group: Pre 55.0 ± 16.0 vs. Post 53.0 ± 12.0, *p* > 0.05 *ES* = −0.14
					Cholesterol – LDL (mg/dl)	Aerobic training group: Pre 129.0 ± 34.0 vs. Post 132.0 ± 35.0, *p* > 0.05 *ES* = 0.09 Aerobic+strength training group: Pre 122.0 ± 21.0 vs. Post 118.0 ± 15.0, *p* > 0.05 *ES* = −0.22
					Cholesterol – Triglycerides (mg/dl)	Aerobic training group: Pre 151.0 ± 65.0 vs. Post 185.0 ± 87.0, *p <* 0.05 *ES* = 0.44 Aerobic+strength training group: Pre 141.0 ± 132.0 vs. Post 135.0 ± 73.0, *p* > 0.05 *ES* = −0.06
					Total cholesterol/HDL ratio	Aerobic training group: Pre 3.8 ± 1.1 vs. Post 3.9 ± 1.1, *p* > 0.05 *ES* = 0.09 Aerobic+strength training group: Pre 3.8 ± 0.8 vs. Post 3.8 ± 0.8, *p* > 0.05 *ES* = 0.00
Marquis et al., 2008 Canada	Randomized controlled trial	16 patients with COPD Irbesartan+exercise group: 10 (7♂; 67.0 ± 7.0 years; FEV_1_ 50.0 ± 19.0%pred; FVC 63.0 ± 16.0%pred; DLCO 80.0 ± 19.0%pred) Placebo+exercise group: 6 (1♂; 72.0 ± 5.0 years; FEV_1_ 39.0 ± 9.0%pred; FVC 63.0 ± 15.0%pred; DLCO 63.0 ± 18.0%pred)	All groups: Aerobic training 80% of WRmax	All groups: 12 weeks 3 sessions/week 30 min/session	Systolic blood pressure at rest (mmHg)	Irbesartan+exercise group: Pre 151.0 ± 19.0 vs. Post 131.0 ± 18.0, *p <* 0.05 *ES* = −1.08 Placebo+exercise group: Pre 140.0 ± 15.0 vs. Post 136.0 ± 15.0, *p* > 0.05 *ES* = −0.27
					Mean systolic blood pressure during 24 h (mmHg)	Irbesartan+exercise group: Pre 135.0 ± 9.0 vs. Post 126.0 ± 12.0, *p <* 0.01 *ES* = −0.85 Placebo+exercise group: Pre 130.0 ± 14.0 vs. Post 128.0 ± 8.0, *p* > 0.05 *ES* = −0.18
					Mean systolic blood pressure at daytime (mmHg)	Irbesartan+exercise group: Pre 139.0 ± 11.0 vs. Post 129.0 ± 15.0, *p <* 0.01 *ES* = −0.76 Placebo+exercise group: Pre 130.0 ± 14.0 vs. Post 131.0 ± 8.0, *p* > 0.05 *ES* = 0.09
					Mean systolic blood pressure at nighttime (mmHg)	Irbesartan+exercise group: Pre 125.0 ± 8.0 vs. Post 121.0 ± 10.0, *p* > 0.05 *ES* = −0.44 Placebo+exercise group: Pre 128.0 ± 16.0 vs. Post 121.0 ± 9.0, *p* > 005 *ES* = −0.54
					Diastolic blood pressure at rest (mmHg)	Irbesartan+exercise group: Pre 78.0 ± 8.0 vs. Post 71.0 ± 10.0, *p <* 0.05 *ES* = −0.77 Placebo+exercise group: Pre 72.0 ± 8.0 vs. Post 68.0 ± 10.0, *p* > 0.05 *ES* = −0.44
					Mean diastolic blood pressure during 24 h (mmHg)	Irbesartan+exercise group: Pre 76.0 ± 9.0 vs. Post 72.0 ± 8.0, *p <* 0.05 *ES* = −0.47 Placebo+exercise group: Pre 70.0 ± 3.0 vs. Post 70.0 ± 8.0, *p* > 0.05 *ES* = 0.00
					Mean diastolic blood pressure at daytime (mmHg)	Irbesartan+exercise group: Pre 80.0 ± 11.0 vs. Post 74.0 ± 10.0, *p <* 0.05 *ES* = −0.84 Placebo+exercise group: Pre 71.0 ± 2.0 vs. Post 72.0 ± 7.0, *p* > 0.05 *ES* = 0.19
					Mean diastolic blood pressure at nighttime (mmHg)	Irbesartan+exercise group: Pre 68.0 ± 6.0 vs. Post 67.0 ± 7.0, *p* > 0.05 *ES* = −0.15 Placebo+exercise group: Pre 66.0 ± 6.0 vs. Post 65.0 ± 8.0, *p* > 0.05 *ES* = −0.14
					Standard deviation of all NN intervals (ms)	Irbesartan+exercise group: Pre 102.0 ± 28.0 vs. Post 144.0 ± 36.0, *p* > 0.05 *ES* = 1.30 Placebo+exercise group: Pre 121.0 ± 27.0 vs. Post 113.0 ± 38.0, *p* > 0.05 *ES* = −0.24
					Adjacent normal-to-normal (NN) intervals differing by more than 50 ms (%)	Irbesartan+exercise group: Pre 9.0 ± 9.0 vs. Post 9.0 ± 8.0, *p* > 0.05 *ES* = 0.00 Placebo+exercise group: Pre 10.0 ± 9.0 vs. Post 10.0 ± 8.0, *p* > 0.05 *ES* = 0.00
					Square root of the mean squared differences of successive NN intervals (ms)	Irbesartan+exercise group: Pre 30.0 ± 12.0 vs. Post 29.0 ± 11.0, *p* > 0.05 *ES* = −0.09 Placebo+exercise group: Pre 31.0 ± 11.0 vs. Post 30.0 ± 10.0, *p* > 0.05 *ES* = −0.10
					Very low frequency (ms)	Irbesartan+exercise group: Pre 3.3 ± 0.2 vs. Post 3.3 ± 0.2, *p* > 0.05 *ES* = 0.00 Placebo+exercise group: Pre 3.1 ± 0.2 vs. Post 3.1 ± 0.5, *p* > 0.05 *ES* = 0.07
					Low frequency (ms)	Irbesartan+exercise group: Pre 2.9 ± 0.3 vs. Post 2.9 ± 0.3, *p* > 0.05 *ES* = −0.03 Placebo+exercise group: Pre 2.8 ± 0.4 vs. Post 2.8 ± 0.4, *p* > 0.05 *ES* = 0.02
					High frequency (ms)	Irbesartan+exercise group: Pre 2.4 ± 0.3 vs. Post 2.4 ± 0.4, *p* > 0.05 *ES* = −0.03 Placebo+exercise group: Pre 2.4 ± 0.4 vs. Post 2.4 ± 0.5, *p* > 0.05 *ES* = −0.12
					Low frequency/High frequency ratio	Irbesartan+exercise group: Pre 3.0 ± 1.3 vs. Post 3.1 ± 1.3, *p* > 0.05 *ES* = 0.08 Placebo+exercise group: Pre 2.1 ± 1.0 vs. Post 2.4 ± 1.0, *p* > 0.05 *ES* = 0.27
Averna et al., 2009 Italy	Randomized controlled trial	56 patients with COPD (29♂; 69.0 ± 5.0 years; FEV_1_ 82.0 ± 16.6%pred; FVC 91.0 ± 17.4%pred)	Aerobic and strength training 40%–50% of HR reserve 50% of 1 RM	12 weeks 3 sessions/week 60 min/session	HR at rest (bpm)	Pre 65.0 ± 10.0 vs. Post 65.0 ± 9.0, p = 0.64 *ES* = 0.00
					Systolic blood pressure at rest (mmHg)	Pre 137.0 ± 12.0 vs. Post 131.0 ± 12.0, p = 0.001 *ES* = −0.70
					Diastolic blood pressure at rest (mmHg)	Pre 84.0 ± 6.0 vs. Post 80.0 ± 7.0, p = 0.001 *ES* = −0.61
Berry et al., 2010 United States of America	Randomized controlled trial	89 patients with COPD (48♂; 66.0 ± 10.0 years; FEV_1_ 53.0 ± 18.5%pred) Arterial hypertension: *n* = 47 Circulatory problems: *n* = 17 Cardiovascular disease: *n* = 39	Aerobic and strength training Dyspnoea 3–5 in the mBorg	12 weeks 3 sessions/week 1 h/session		
Rejbi et al., 2010 Tunisia	Non-randomized controlled trial	26 patients with COPD (61.0 ± 4.0 years; FEV_1_ 48.9 ± 11.3%pred; FVC 58.8 ± 9.8%pred)	Pulmonary rehabilitation HR of the gas exchange threshold	3 months 3 sessions/week 45 min/session	HR at rest (bpm)	Pre 75.6 ± 13.9 vs. Post 76.5 ± 14.0, *p* > 0.05 *ES* = 0.06
Camillo et al., 2011 Brazil	Randomized controlled trial	40 patients with COPD High-intensity group: 20 (10♂; 67.0 ± 7.0 years; FEV_1_ 40.0 ± 13.0%pred) Low-intensity group: 20 (11♂; 65.0 ± 10.0 years; FEV_1_ 39.0 ± 14.0%pred)	High-intensity group: Aerobic and strength training 60% of WRmax 75% of average walking speed in the 6MWT 70% of 1RM Low-intensity group: Strength training	All groups: 12 weeks 3 sessions/week 1 h/session	Standard deviation of N-N intervals (ms)	High-intensity group: Pre 29.0 ± 15.0 vs. Post 36.0 ± 19.0, *p <* 0.05 *ES* = 0.41 Low-intensity group: Pre 25.0 ± 12.0 vs. Post 22.0 ± 10.0, *p* > 0.05 *ES* = −0.27
					Square root of the mean squared difference of the successive N-N intervals (ms)	High-intensity group: Pre 22.0 ± 14.0 vs. Post 28.0 ± 22.0, *p <* 0.05 *ES* = 0.33 Low-intensity group: Pre 22.0 ± 22.0 vs. Post 19.0 ± 14.0, *p* > 0.05 *ES* = −0.16
					Low frequency in supine (%)	High-intensity group: Pre 44.0 ± 15.0 vs. Post 42.0 ± 24.0, *p* > 0.05 *ES* = −0.10 Low-intensity group: Pre 48.0 ± 19.0 vs. Post 43.0 ± 19.0, *p* > 0.05 *ES* = −0.26
					Low frequency in orthostatic (%)	High-intensity group: Pre 55.0 ± 21.0 vs. Post 50.0 ± 20.0, *p* > 0.05 *ES* = −0.24 Low-intensity group: Pre 58.0 ± 15.0 vs. Post 62.0 ± 20.0, *p* > 0.05 *ES* = 0.23
					High frequency in supine (%)	High-intensity group: Pre 56.0 ± 15.0 vs. Post 58.0 ± 24.0, *p* > 0.05 *ES* = 0.10 Low-intensity group: Pre 51.0 ± 19.0 vs. Post 56.0 ± 19.0, *p* > 0.05 *ES* = 0.26
					High frequency in orthostatic (%)	High-intensity group: Pre 44.0 ± 21.0 vs. Post 50.0 ± 20.0, *p* > 0.05 *ES* = 0.29 Low-intensity group: Pre 41.0 ± 15.0 vs. Post 37.0 ± 20.0, *p* > 0.05 *ES* = −0.23
					Low frequency/High frequency ratio in supine	High-intensity group: Pre 0.9 ± 0.8 vs. Post 1.3 ± 1.5, *p* > 0.05 *ES* = 0.60 Low-intensity group: Pre 1.2 ± 0.9 vs. Post 1.1 ± 1.2, *p* > 0.05 *ES* = −0.09
					Low frequency/High frequency ratio in orthostatic	High-intensity group: Pre 2.3 ± 3.1 vs. Post 1.3 ± 0.9, *p* > 0.05 *ES* = −0.44 Low-intensity group: Pre 1.7 ± 1.0 vs. Post 2.8 ± 2.8, *p* > 0.05 *ES* = 0.52
Lan et al., 2011 Taiwan	Non-controlled study	44 patients with COPD Underweight group: 22 (21♂; 69.1 ± 12.0 years; FEV_1_ 52.8 ± 17.1%pred; FVC 79.5 ± 21.4%pred) Non-underweight group: 22 (21♂; 71.4 ± 7.5 years; FEV_1_ 51.5 ± 13.3%pred; FVC 79.1 ± 15.1%pred)	All groups: Pulmonary rehabilitation 50%–75% of VO2peak	All groups: 12 weeks 2 sessions/week 40–50 min/session	HR at rest (bpm)	Underweight group: Pre 85.2 ± 13.0 vs. Post 83.1 ± 11.7, p = 0.315 *ES* = −0.17 Non-underweight group: Pre 88.2 ± 11.6 vs. Post 86.0 ± 10.8, p = 0.029 *ES* = −0.20
Corhay et al., 2012 Belgium	Non-controlled study	140 patients with COPD <65 years group: 69 (42♂; 57.6 ± 5.2 years; FEV_1_ 38.1 ± 10.8%pred) Cardiovascular disease: *n* = 19 65–74 years group: 50 (36♂; 69.5 ± 2.6 years; FEV_1_ 39.5 ± 11.7%pred) Cardiovascular disease: *n* = 23 ≥75 years group: 21 (17♂; 77.4 ± 2.5 years; FEV_1_ 39.9 ± 9.2%pred) Cardiovascular disease: *n* = 14	All groups: Pulmonary rehabilitation 50%–80% of WRmax 60% of maximal walking speed in the 6MWT 50% of 1RM	All groups: 6 months 2–3 sessions/week 2 h/session		
Georgiopoulou et al., 2012 Greece	Pre-Post study	45 patients with COPD (40♂; 66.5 ± 7.6 years; FEV_1_ 45.7 ± 18.7%pred; FVC 78.3 ± 18.6%pred)	Pulmonary rehabilitation 60%–80% of WRmax	12 weeks 3 sessions/week 40 min/session	HR at rest (bpm)	Pre 88.0 ± 10.7 vs. Post 83.3 ± 10.5, p = 0.004 *ES* = −0.63
					HR recovery (bpm)	Pre 16.2 ± 8.0 vs. Post 18.4 ± 8.4, p = 0.01 *ES* = 0.27
Lan et al., 2013 Taiwan	Pre-Post study	26 patients with COPD (71.0 ± 10.7 years; FEV_1_ 64.8 ± 23.0%pred; FVC 88.3 ± 34.5%pred)	Pulmonary rehabilitation 75%–100% of VO2max	12 weeks 2 sessions/week 40 min/session	HR (bpm)	Pre 134.5 ± 14.9 vs. Post 137.4 ± 19.9, p = 0.36 *ES* = 0.16
					Mean blood pressure (mmHg)	Pre 109.6 ± 15.7 vs. Post 110.3 ± 15.1, p = 0.72 *ES* = 0.05
					Oxygen pulse (ml/beat)	Pre 9.2 ± 2.5 vs. Post 9.8 ± 2.7, p = 0.02 *ES* = 0.23
Cheng et al., 2014 Taiwan	Pre-Post study	64 patients with COPD (55♂; 70.1 ± 8.7 years; FEV_1_ 44.9 ± 11.7%pred; FVC 78.2 ± 17.4%pred)	Pulmonary rehabilitation 60%–100% of VO2peak	12 weeks 2 sessions/week 50 min/session	HR at rest (bpm)	Pre 87.2 ± 12.7 vs. Post 83.9 ± 13.5, p = 0.048 *ES* = −0.25
					Oxygen pulse (ml/beat)	Pre 7.2 ± 1.9 vs. Post 7.9 ± 2.2, p = 0.005 *ES* = 0.34
					Oxygen pulse (%)	Pre 76.8 ± 18.4 vs. Post 85.2 ± 24.8, p = 0.003 *ES* = 0.38
					Standard deviation of N-N	At rest: Pre vs. Post, *p <* 0.05 At exercise: Pre vs. Post, *p <* 0.05
					Square root of the mean sum of the squares of the difference between adjacent normal R-R intervals	At rest: Pre vs. Post, *p <* 0.05 At exercise: Pre vs. Post, *p <* 0.05
					Low frequency	At rest: Pre vs. Post, *p <* 0.05 At exercise: Pre vs. Post, *p <* 0.05
					High frequency	At rest: Pre vs. Post, *p <* 0.05 At exercise: Pre vs. Post, *p <* 0.05
					Low frequency/High frequency ratio	At rest: Pre vs. Post, *p <* 0.05 At exercise: Pre vs. Post, *p <* 0.05
Borghi-Silva et al., 2015 Brazil	Randomized controlled trial	10 patients with COPD (7♂; 67.0 ± 7.0 years; FEV_1_ 32.0 ± 11.0%pred; FVC 58.0 ± 15.0%pred)	Aerobic training 70% of peak speed in CPET	12 weeks 3 sessions/week 30 min/session	Mean of RR and its standard deviation at rest (ms)	Pre 17.2 ± 7.3 vs. Post 25.4 ± 5.5, *p <* 0.05 *ES* = 1.27
					Mean of RR and its standard deviation at constant speed (ms)	Pre 12.7 ± 5.1 vs. Post 18.3 ± 4.7, *p* > 0.05 *ES* = 1.14
					Square root of the mean squared differences of successive RRi at rest (ms)	Pre 11.7 ± 6.0 vs. Post 22.9 ± 0.2, *p <* 0.05 *ES* = 2.64
					Square root of the mean squared differences of successive RRi at constant speed (ms)	Pre 3.5 ± 1.7 vs. Post 16.9 ± 7.0, *p <* 0.05 *ES* = 2.63
					Nonlinear indices – SD1 at rest	Pre 7.1 ± 4.2 vs. Post 19.2 ± 11.8, *p <* 0.05 *ES* = 1.37
					Nonlinear indices – SD1 at constant speed	Pre 3.7 ± 1.7 vs. Post 13.6 ± 8.8, *p <* 0.05 *ES* = 1.56
					Nonlinear indices – SD2 at rest	Pre 31.2 ± 6.6 vs. Post 46.1 ± 22.0, *p <* 0.05 *ES* = 0.92
					Nonlinear indices – SD2 at constant speed	Pre 17.3 ± 5.9 vs. Post 25.4 ± 6.5, *p <* 0.05 *ES* = 1.30
					Low frequency (nu)	Pre 0.6 ± 0.2 vs. Post 0.5 ± 0.2, *p* > 0.05 *ES* = −0.60
					High frequency (nu)	Pre 0.4 ± 0.2 vs. Post 0.5 ± 0.2, *p* > 0.05 *ES* = 0.60
					Low frequency/High frequency ratio	Pre 2.4 ± 2.3 vs. Post 1.8 ± 1.7, *p* > 0.05 *ES* = −0.31
					Sample entropy	Pre 0.7 ± 0.2 vs. Post 0.9 ± 0.2, *p* > 0.05 *ES* = 1.03
Campos et al., 2015 Chile	Pre-Post study	39 patients with COPD (36%♂; 67.3 ± 8.5 years; FEV_1_ 59.8 ± 21.0%pred; FVC 78.0 ± 20.3%pred) Arterial hypertension: *n* = 31 Dyslipidemia: *n* = 5 Congestive heart failure: *n* = 3	Pulmonary rehabilitation 70%–80% of 6MWT	12 weeks 2 sessions/week 90 min/session		
Leite et al., 2015 Brazil	Non-randomized controlled trial	10 patients with COPD (62.0 (60.3; 69.3) years; FEV_1_ 55.0 (39.0; 70.0)%pred; FVC 78.0 (66.3; 83.5)%pred)	Aerobic training 60%–100% of VO2peak	12 weeks 3 sessions/week 20–50 min/session	Standard deviation of the mean of all normal RR intervals (ms)	Pre 19.8 ± 6.2 vs. Post 24.9 ± 8.6, *p* > 0.05 *ES* = 0.67
					Root mean square of differences between adjacent normal RR intervals in a time interval (ms)	Pre 14.2 ± 5.7 vs. Post 18.3 ± 6.2, *p* > 0.05 *ES* = 0.69
					Spectral component of low frequency (ms2)	Pre146.1 ± 118.9 vs. Post 177.7 ± 125.6, *p* > 0.05 *ES* = 0.26
					Spectral component of low frequency (nu)	Pre 67.5 ± 16.0 vs. Post 58.5 ± 13.6, *p* > 0.05 *ES* = −0.61
					Spectral component of high frequency (ms2)	Pre 62.3 ± 46.8 vs. Post 113.2 ± 62.2, *p <* 0.05 *ES* = 0.92
					Spectral component of high frequency (nu)	Pre 32.6 ± 15.9 vs. Post 41.5 ± 13.6, *p* > 0.05 *ES* = 0.60
					Low frequency/High frequency ratio	Pre 2.9 ± 2.2 vs. Post 1.6 ± 0.8, *p* > 0.05 *ES* = −0.78
Mkacher et al., 2015 Tunisia	Randomized controlled trial	68 patients with COPD Pulmonary rehabilitation group: 33 (33♂; 61.2 ± 3.2 years; FEV_1_ 38.6 ± 8.6%pred) Pulmonary rehabilitation+balance group: 35 (35♂; 58.3 ± 4.3 years; FEV_1_ 39.4 ± 10.3%pred)	All groups: Pulmonary rehabilitation	All groups: 6 months 6 sessions/week (3 times/week, 2 sessions/day)	HR at rest (bpm)	Pulmonary rehabilitation group: Pre 72.7 ± 8.9 vs. Post 73.0 ± 4.3, *p* > 0.05 *ES* = 0.04 Pulmonary rehabilitation+balance group: Pre 75.3 ± 3.9 vs. Post 73.5 ± 4.5, *p* > 0.05 *ES* = −0.43
Spielmanns et al., 2015 Germany	Randomized controlled trial	36 patients with COPD Compressed air group: 17 (64.0 ± 8.4 years; FEV_1_ 43.0 ± 12.0%pred) Arterial hypertension: *n* = 7 Cardiovascular disease: *n* = 2 Oxygen group: 19 (65.0 ± 8.7 years; FEV_1_ 44.0 ± 10.0%pred) Arterial hypertension: *n* = 8 Cardiovascular disease: *n* = 4	All groups: Continuous aerobic training 70%–85% of WRmax Interval aerobic training 110%–125% of WRmax	All groups: 24 weeks 3 sessions/week 30 min/session		
Cardoso et al., 2016 Brazil	Non-randomized controlled trial	10 patients with COPD (65.2 ± 4.2 years; FEV_1_ 41.8 ± 21.3%pred; FVC 60.7 ± 18.0%pred) Arterial hypertension: *n* = 7	Pulmonary rehabilitation 75% of WRmax 60% of 1RM	12 weeks 3 sessions/week >30 min/session		
Engel et al., 2016 Australia	Randomized controlled trial	33 patients with COPD (10♂; 65.5 ± 4.0 years; FEV_1_ 1.6 ± 0.5 L; FVC 2.3 ± 0.7 L)	Pulmonary rehabilitation	16 weeks	Systolic blood pressure (mmHg)	Mean Pre/Post difference Group 1: −3.6, 95%CI (−13.5; 6.3) Group 2: −10.6, 95%CI (−19.6; −1.5) Group 3: −8.3, 95%CI (−20.5; 3.8)
					Diastolic blood pressure (mmHg)	Mean Pre/Post difference Group 1: −3.5, 95%CI (−12.6; 5.6) Group 2: −7.7, 95%CI (−17.1; 1.8) Group 3: −4.7, 95%CI (−13.5; 4.2)
Boeselt et al., 2017 Germany	Non-randomized controlled trial	20 patients with COPD (16♂; 65.9 ± 8.2 years; FEV_1_ 67.9 ± 29.2%pred) Arterial hypertension: *n* = 5 Cardiovascular disease: *n* = 2	Strength training 35%–75% of 1RM	3 months 2 sessions/week 90 min/session		
Kanao et al., 2017 Japan	Pre-Post study	29 patients with COPD (26♂; 73.2 ± 5 years; FEV_1_ 51.0 ± 121.3%pred) Arterial hypertension: *n* = 10 Cardiovascular disease: *n* = 5	Pulmonary rehabilitation 60% of WRpeak	12 weeks 2 sessions/week		
Pacheco et al., 2017 Spain	Observational study	35 patients with COPD (88.6%♂; 65.1 ± 9.0 years; FEV_1_ 42.2 ± 10.5; FVC 67.8 ± 13.3%pred; DLCO 47.9 ± 21.0%pred Arterial hypertension: *n* = 20 Dyslipidemia: *n* = 6 Congestive heart failure: *n* = 7 Ischemic cardiomyopathy: *n* = 3	Pulmonary rehabilitation 70% of WRmax 75% of 1RM	12 weeks 3 sessions/week >30 min/session		
Papp et al., 2017 Sweden	Randomized controlled trial	17 patients with COPD (7♂; 69.0 (62.0; 72.1) years; FEV_1_ 64.3 ± 15.4%pred)	Aerobic and strength training 70% of 1RM Perceived exertion 12–14 in the Borg	12 weeks 2 sessions/week 60–70 min/session	HR at rest (bpm)	Mean Pre/Post difference 0.6, p = 0.82
					Systolic blood pressure at rest (mmHg)	Mean Pre/Post difference 4.2
					Diastolic blood pressure at rest (mmHg)	Mean Pre/Post difference 5.7, p = 0.04
					Number of pairs of adjacent NN intervals differing by more than 50 ms in the 5 min recording divided by the total number of all NN intervals (%)	Mean Pre/Post difference 0.6, p = 0.56
					Square root of the mean of the sum of the squares of differences between adjacent NN intervals	Mean Pre/Post difference −3.2, p = 0.27
Vasilopoulou et al., 2017 Greece	Randomized controlled trial	50 patients with COPD (38♂; 66.7 ± 7.3 years; FEV_1_ 51.8 ± 17.3%pred; FVC 78.4 ± 18.4%pred; DLCO 57.0 ± 20.4%pred) Cardiovascular disease: *n* = 15	Pulmonary rehabilitation	12 months 2 sessions/week		
Lan et al., 2018 Taiwan	Pre-Post study	43 patients with COPD (31♂; 69.7 ± 8.8 years; FEV_1_ 49.5 ± 19.9%pred; FVC 76.5 ± 22.3%pred)	Pulmonary rehabilitation	12 weeks 2 sessions/week 40 min/session	HR at rest	Pre vs. Post *p* > 0.05
					Mean blood pressure at rest	Pre vs. Post *p <* 0.05
					Oxygen pulse	Pre vs. Post *p <* 0.05
Moezy et al., 2018 Iran	Randomized controlled trial	14 patients with COPD (71.4%♂; 64.7 ± 7.5 years; FEV_1_ 60.2 ± 14.0%pred)	Aerobic training Dyspnoea 3–4 in the mBorg	12 weeks 3 sessions/week 15–60 min/session	HR at rest (bpm)	Pre 80.4 ± 12.6 vs. Post 77.8 ± 11.9, p = 0.968 *ES* = −0.21
Silva et al., 2018 Brazil	Randomized controlled trial	48 patients with COPD Elastic resistances group: 32 (69.4 ± 9.0 years; FEV_1_ 50.7 ± 16.7%pred; FVC 72.5 ± 13.2%pred) Weight machines group: 16 (64.9 ± 11.2 years; FEV_1_ 45.4 ± 15.2%pred; FVC 66.1 ± 14.0%pred)	All groups: Strength training	All groups: 12 weeks 3 sessions/week 60 min/session	Total cholesterol (mg/dL)	Elastic resistances group: Pre 108.4 ± 25.3 vs. Post 104.6 ± 14.3, *p* > 0.05 *ES* = −0.18 Weight machines group: Pre 84.6 ± 27.0 vs. Post 71.1 ± 32.0, *p* > 0.05 *ES* = −0.46
					Cholesterol – HDL (mg/dL)	Elastic resistances group: Pre 58.4 ± 23.2 vs. Post 63.4 ± 17.3, *p* > 0.05 *ES* = 0.24 Weight machines group: Pre 132.3 ± 43.6 vs. Post 150.3 ± 52.3, *p* > 0.05 *ES* = 0.37
					Cholesterol – Triglycerides (mg/dL)	Elastic resistances group: Pre 154.2 ± 62.3 vs. Post 129.7 ± 40.3, *p* > 0.05 *ES* = −0.47 Weight machines group: Pre 104.8 ± 38.4 vs. Post 99.9 ± 32.9, *p* > 0.05 *ES* = −0.14
					Total cholesterol/HDL ratio (mg/dL)	Elastic resistances group: Pre 50.7 ± 39.9 vs. Post 40.9 ± 25.8, *p* > 0.05 *ES* = −0.29 Weight machines group: Pre 71.9 ± 31.2 vs. Post 61.3 ± 15.4, *p* > 0.05 *ES* = −0.43
Charikiopoulou et al., 2019 Greece	Non-controlled study	32 patients with COPD (25♂; 66.0 ± 6.0 years; FEV_1_ 43.1 ± 15.1%pred; DLCO 38.2 ± 22.8%pred) Cardiovascular disease: *n* = 22	Pulmonary rehabilitation 100% of WRmax	13 weeks 2 sessions/week ≥1 h/session		
Mekki et al., 2019 Tunisia	Randomized controlled trial	45 patients with COPD Pulmonary rehabilitation+NMES group: 25 (25♂; 59.6 ± 4.8 years; FEV_1_ 57.7 ± 14.4%pred; FVC 76.0 ± 13.2%pred) Pulmonary rehabilitation group: 20 (20♂; 59.5 ± 3.1 years; FEV_1_ 57.1 ± 10.2%pred; FVC 75.9 ± 7.8%pred)	All groups: Pulmonary rehabilitation 60%–70% of HRmax in the 6MWT 50%–85% of 10RM	All groups: 6 months 3 sessions/week 80 min/session	HR at rest (bpm)	Pulmonary rehabilitation+NMES group: Pre 80.0 ± 9.0 vs. Post 78.0 ± 9.0, *p <* 0.001 *ES* = −0.22 Pulmonary rehabilitation group: Pre 80.0 ± 7.0 vs. Post 77.0 ± 7.0, *p <* 0.001 *ES* = −0.43
Silva et al., 2019 Brazil	Randomized controlled trial	19 patients with COPD Elastic resistances group: 9 (65.9 ± 8.9 years; FEV_1_ 45.2 ± 16.2%pred; FVC 64.7 ± 19.0%pred) Weight machines group: 10 (65.5 ± 9.8 years; FEV_1_ 57.6 ± 16.3%pred; FVC 79.8 ± 11.5%pred)	All groups: Strength training	All groups: 12 weeks 3 sessions/week 60 min/session	HR (bpm)	Elastic resistances group: Pre 74.1 ± 8.8 vs. Post 76.8 ± 8.9, *p* > 0.05 *ES* = 0.30 Weight machines group: Pre 71.4 ± 6.4 vs. Post 68.9 ± 9.9, *p* > 0.05 *ES* = −0.30
					Systolic blood pressure (mmHg)	Elastic resistances group: Pre 120.0 (105.0; 135.0) vs. Post 120.0 (110.0; 120.0), *p* > 0.05 Weight machines group: Pre 120.0 (117.5; 130.0) vs. Post 120.0 (110.0; 120.0), *p* > 0.05
					Diastolic blood pressure (mmHg)	Elastic resistances group: Pre 70.0 (70.0; 80.0) vs. Post 70.0 (70.0; 75.0), *p* > 0.05 Weight machines group: Pre 80.0 (70.0; 90.0) vs. Post 75.0 (67.5; 80.0), *p* > 0.05

Data are presented as mean ± standard deviation or median (interquartile range), unless otherwise stated. Legend: 6MWT, 6-min walk test; 12MWT, 12-min walk test; 1RM, one repetition maximum; 10RM, ten repetition maximum; 15RM, fifteen repetition maximum; 95%CI, 95% confidence interval; %pred, percentage predicted; COPD, chronic obstructive pulmonary disease; CPET, cardiopulmonary exercise test; DLCO, diffusing capacity for carbon monoxide; ES, effect size; FEV_1_, forced expiratory volume in 1 s; FVC, forced vital capacity; HDL, high density lipoprotein; HR, heart rate; HR_max_, maximum heart rate; ILD, interstitial lung disease; IPF, idiopathic pulmonary fibrosis; LDL, low density lipoprotein; mBorg, modified Borg scale; NMES, neuromuscular electrical stimulation; VO_2_max, maximal oxygen uptake; VO_2_peak, peak oxygen uptake; WR, work rate; WR_max_, maximal work rate; WR_peak_, peak work rate.

**Table 3:** Characteristics of the studies in patients with asthma included in phase 2 (i.e., studies that specified the prevalence of cardiovascular comorbidities in the baseline characteristics of the population under study and/or reported at least one cardiovascular outcome) (*n* = 2).

Study and Country	Study design	Population	Intervention	Duration and Frequency	Cardiovascular Outcomes and Outcome measures	Results on Cardiovascular Outcomes
Cochrane et al., 1990 Scotland	Randomized controlled trial	18 patients with Asthma (27.0 ± 17.0 years; FEV_1_ 76.0 ± 12.0%pred)	Aerobic and muscle strength training 75% of HRmax	3 months 3 sessions/week 30 min/session	Oxygen pulse (mL/beat)	Pre 8.8 ± 2.3 vs. Post 10.8 ± 2.4, *p <* 0.001 *ES* = 0.85
					Total blood cholesterol (mmol/L)	Pre 5.4 ± 1.1 vs. Post 5.3 ± 1.1, *p* > 0.05 *ES* = −0.09
					Cholesterol – HDL (mmol/L)	Pre 1.7 ± 0.4 vs. Post 1.6 ± 0.3, *p* > 0.05 *ES* = −0.28
					Cholesterol – LDL (mmol/L)	Pre 3.2 ± 1.2 vs. Post 2.9 ± 0.9, *p* > 0.05 *ES* = −0.28
El-Kader et al., 2016 Saudi Arabia	Randomized controlled trial	40 patients with Asthma (23♂; 47.2 ± 6.5 years; FEV_1_ 1.4 ± 0.7 L)	Aerobic training 60%–80% of HRmax	6 months 3 sessions/week 30 min/session	Cholesterol – HDL (mg/dL)	Pre 34.7 ± 5.6 vs. Post 37.9 ± 4.6, *p <* 0.05 *ES* = 0.62
					Cholesterol – LDL (mg/dL)	Pre 133.7 ± 13.2 vs. Post 120.3 ± 11.5, *p <* 0.05 *ES* = −1.08
					Cholesterol – Triglycerides (mg/dL)	Pre 155.4 ± 12.6 vs. Post 127.7 ± 11.3, *p <* 0.05 *ES* = −2.31

Data are presented as mean ± standard deviation, unless otherwise stated. Legend: %pred, percentage predicted; ES, effect size; FEV_1_, forced expiratory volume in 1 s; HDL, high density lipoprotein; HR, heart rate; HR_max_, maximum heart rate; LDL, low density lipoprotein.

**Table 4:** Characteristics of the studies in patients with ILD included in phase 2 (i.e., studies that specified the prevalence of cardiovascular comorbidities in the baseline characteristics of the population under study and/or reported at least one cardiovascular outcome) (*n* = 8).

Study and Country	Study Design	Population	Intervention	Duration and Frequency	Cardiovascular Outcomes and Outcome Measures	Results on Cardiovascular Outcomes
Gaunaurd et al., 2014 United States of America	Randomized controlled trial	11 patients with IPF (71.0 ± 6.0 years; FVC 60.0 ± 11.0%pred; DLCO 44.0 ± 11.0%pred) History of heart disease: *n* = 1	Pulmonary rehabilitation 70%–80% of HRmax	12 weeks 2 sessions/week 90 min/session		
Vainshelboim et al., 2014 Israel	Randomized controlled trial	15 patients with IPF (10♂; 68.8 ± 6 years; FEV_1_ 68.5 ± 15.8%pred; FVC 66.1 ± 14.8%pred; DLCO 48.6 ± 17.2%pred) Arterial hypertension: *n* = 12 Coronary heart disease: *n* = 7 Pulmonary hypertension: *n* = 5	Aerobic and strength training 50%–70% of WRpeak 70%–90% of average walking speed in the 6MWT 3–6 in the mBorg	12 weeks 2 sessions/week 60 min/session	HR at rest (bpm)	Mean Pre/Post difference −2.4 ± 9.1 *ES* = −0.26
					Systolic blood pressure at rest (mmHg)	Mean Pre/Post difference −2.9 ± 13.6 *ES* = −0.21
					Diastolic blood pressure at rest (mmHg)	Mean Pre/Post difference 1.5 ± 7.1 *ES* = 0.21
					Oxygen pulse (ml/beat)	Mean Pre/Post difference 0.9 ± 1.5 *ES* = 0.62
Marcellis et al., 2015 The Netherlands	Pre-Post study	18 patients with Sarcoidosis (14♂; 50.3 ± 10.4 years; FEV_1_ 93.6 ± 17.0%pred; FVC 102.2 ± 18.1%pred; DLCO 91.2 ± 18.4%pred)	Aerobic and strength training 40% of 1RM 60% of maximal walking speed in the 6MWT 50% of WRmax	13 weeks 3 sessions/week 1 h/session	HR at rest (bpm)	Pre 82.7 ± 13.1 vs. Post 77.1 ± 12.8, p = 0.11 *ES* = −0.43
Vainshelboim at al., 2015 Israel	Randomized controlled trial	15 patients with IPF (10♂; 68.8 ± 6 years; FVC 66.1 ± 14.8%pred; DLCO 48.6 ± 17.2%pred) Arterial hypertension: *n* = 12 Coronary heart disease: *n* = 7 Pulmonary hypertension: *n* = 5	Aerobic and strength training 50%–70% of WRpeak 70%–90% of average walking speed in the 6MWT Perceived exertion 3–6 in the mBorg	12 weeks 2 sessions/week 60 min/session		
Boström et al., 2016 Sweden	Randomized controlled trial	18 patients with Systemic lupus erythematosus (0♂; 52.0 ± 10.0 years) Arterial hypertension: *n* = 6	Pulmonary rehabilitation 65%–80% of HRmax Perceived exertion 13–16 in the Borg	12 weeks 2 sessions/week 60 min/session	HR at rest Blood pressure at rest	Pre vs. Post, p = 0.04 Pre vs. Post, *p* > 0.05
Vainshelboim et al., 2017 Israel	Randomized controlled trial	15 patients with IPF (10♂; 68.8 ± 6.0 years; FVC 66.1 ± 14.8%pred; DLCO 48.6 ± 17.2%pred) Arterial hypertension: *n* = 12 Coronary heart disease: *n* = 7 Pulmonary hypertension: *n* = 5	Aerobic and strength training 50%–70% of WRpeak 70%–90% of average walking speed in the 6MWT	12 weeks 2 sessions/week 60 min/session	HR (bpm)	Mean Pre/Post difference −2.4 ± 9.1 *ES* = −0.26
					HR reserve (bpm)	Mean Pre/Post difference 6.7 ± 11.0 *ES* = 0.61
					Systolic blood pressure (mmHg)	Mean Pre/Post difference −2.9 ± 13.6 *ES* = −0.21
					Diastolic blood pressure (mmHg)	Mean Pre/Post difference 1.5 ± 7.1 *ES* = 0.21
					Rate pressure product (bpm/mmHg)	Mean Pre/Post difference 1685.0 ± 3338.0 *ES* = 0.50
					Left atrium diameter (cm)	Mean Pre/Post difference 0.0 ± 0.5 *ES* = 0.04
					Left atrium area (cm2)	Mean Pre/Post difference 0.2 ± 2.7 *ES* = 0.07
					Left ventricle posterior wall thickness (cm)	Mean Pre/Post difference 0.0 ± 0.1 *ES* = 0.30
					Intra-ventricular septum thickness (cm)	Mean Pre/Post difference 0.1 ± 0.1 *ES* = 0.60
					Left ventricle end systolic diameter index (cm/m2)	Mean Pre/Post difference −0.1 ± 0.3 *ES* = −0.40
					Left ventricle end diastolic diameter index (cm/m2)	Mean Pre/Post difference −0.1 ± 0.3 *ES* = −0.47
					Stroke volume (mL/beat)	Mean Pre/Post difference −4.5 ± 13.4 *ES* = −0.34
					Cardiac output (L/min)	Mean Pre/Post difference −0.4 ± 0.8 *ES* = −0.50
					Cardiac index (L/min/m2)	Mean Pre/Post difference −0.2 ± 0.4 *ES* = −0.50
					Ejection fraction (%)	Mean Pre/Post difference 0.8 ± 3.0 *ES* = 0.27
					Fractioning shortening (%)	Mean Pre/Post difference 0.9 ± 6.2 *ES* = 0.15
					Earlier transmitral velocity (E) (ms)	Mean Pre/Post difference 0.8 ± 16.9 *ES* = 0.05
					Late trasmitral velocity (A) (ms)	Mean Pre/Post difference 5.1 ± 20.7 *ES* = 0.25
					E/A ratio	Mean Pre/Post difference 0.0 ± 0.4 *ES* = 0.00
					Isovolumic relaxation time (ms)	Mean Pre/Post difference 9.1 ± 32.1 *ES* = 0.28
					Deceleration time (ms)	Mean Pre/Post difference 11.0 ± 52.7 *ES* = 0.21
					Systolic pulmonary arterial pressure (mmHg)	Mean Pre/Post difference −0.5 ± 6.8 *ES* = −0.07
					Peak circulatory power (mLO2/kg/min/mmHg)	Mean Pre/Post difference 490.0 ± 637.0 *ES* = 0.77
					Peak cardiac power output (W)	Mean Pre/Post difference 0.3 ± 0.3 *ES* = 0.94
					Peak stroke work (mLO2/beat/mmHg)	Mean Pre/Post difference 221.0 ± 343.0 *ES* = 0.64
Naz et al., 2018a Turkey	Pre-Post study	14 patients with ILD (5♂; 63.0 (53.0; 70.0) years; FEV_1_ 78.0 (69.0; 83.0)%pred; FVC 74.0 (67.0; 78.0)%pred; DLCO 40.0 (19.0; 45.0)%pred) Arterial hypertension: *n* = 7 Congestive heart failure: *n* = 2	Aerobic and strength training 80% of peak walking speed in the 6MWT 70% of WRmax Dyspnoea and perceived exertion 4–6 in the mBorg	12 weeks 2 sessions/week 60–90 min/session		
Naz et al., 2018b Turkey	Randomized controlled trial	9 patients with Sarcoidosis (33.3%♂; 59.0 (52.0; 64.0) years; FEV_1_ 73.0 (65.0; 85.0)%pred; FVC 76.0 (66.0; 90.0)%pred; DLCO 45.0 (36.0; 54.0)%pred)	Aerobic and strength training 80% of the peak speed in the 6MWT Fatigue 4–6 in the mBorg	12 weeks 2 sessions/week	HR (bpm)	Median Pre/Post difference 0.0 [−6.0; 5.0], *p* > 0.05

Data are presented as mean ± standard deviation or median (interquartile range), unless otherwise stated. Legend: 6MWT, 6-min walk test; 1RM, one repetition maximum; %pred, percentage predicted; DLCO, diffusing capacity for carbon monoxide; ES, effect size; FEV_1_, forced expiratory volume in 1 s; FVC, forced vital capacity; HR, heart rate; HR_max_, maximum heart rate; ILD, interstitial lung disease; IPF, idiopathic pulmonary fibrosis; mBorg, modified Borg scale; WR, work rate; WR_max_, maximal work rate; WR_peak_, peak work rate.

## Appendix A. Search Strategy

### A1. PubMed

**#1** Search (“chronic obstructive pulmonary disease” OR “copd” OR “asthma” OR “interstitial lung diseas*” OR “ILD" OR "parenchymal lung disease" OR "parenchymal lung disorder" OR "pulmonary fibrosis" OR “sarcoidosis” OR "interstitial pneumonia" OR "connective tissue disease" OR "collagen vascular disease" OR "occupational lung disease" OR "hypersensitivity pneumonitis" OR “asbestosis” OR “silicosis” OR “beryliosis” OR "respiratory bronchiolitis" OR "desquamative interstitial pneumonia" OR "cryptogenic organising pneumonia" OR "lymphoid interstitial pneumonia" OR "pleuroparenchymal fibroelastosis" OR “pneumoconiosis” OR "extrinsic allergic alveolitis" OR "Iatrogenic interstitial lung disease" OR "post-infectious interstitial lung disease" OR “granulomatose” OR "systemic sclerosis" OR “polymyositis” OR “dermatomyositis” OR "systemic lupus erythematosus" OR "Hamman-Rich syndrome" OR “bagassosis” OR “histiocytosis” OR "fibrotic interstitial lung disease" OR "fibrotic lung disease")

**#2** Search (“exercise” OR “walking” OR “aerobic training” OR “endurance training” OR “interval training” OR “high-intensity training” OR “resistance training” OR “strength training”)

**#3** Search ("Pulmonary Disease, Chronic Obstructive” OR “Asthma” OR “Lung Diseases, Interstitial” OR “Pulmonary Fibrosis” OR “Sarcoidosis” OR “Connective Tissue Diseases” OR “Alveolitis, Extrinsic Allergic” OR “Asbestosis” OR “Silicosis” OR “Pneumoconiosis” OR “Scleroderma, Systemic” OR “Polymyositis” OR “Dermatomyositis” OR “Lupus Erythematosus, Systemic” OR “Histiocytosis”))

**#4** Search (“Exercise” OR “Walking” OR “Endurance Training” OR “Resistance Training”)

**#5** Search (#1 AND #2 [Title/Abstract]) OR (#3 AND #4 [MeSH Terms])

### A2. Cochrane, Scopus and Web of Science

**#1** Search (“chronic obstructive pulmonary disease” OR copd OR asthma OR “interstitial lung diseas*” OR ILD OR "parenchymal lung disease" OR "parenchymal lung disorder" OR "pulmonary fibrosis" OR sarcoidosis OR "interstitial pneumonia" OR "connective tissue disease" OR "collagen vascular disease" OR "occupational lung disease" OR "hypersensitivity pneumonitis" OR asbestosis OR silicosis OR beryliosis OR "respiratory bronchiolitis" OR "desquamative interstitial pneumonia" OR "cryptogenic organising pneumonia" OR "lymphoid interstitial pneumonia" OR "pleuroparenchymal fibroelastosis" OR pneumoconiosis OR "extrinsic allergic alveolitis" OR "Iatrogenic interstitial lung disease" OR "post-infectious interstitial lung disease" OR granulomatose OR "systemic sclerosis" OR polymyositis OR dermatomyositis OR "systemic lupus erythematosus" OR "Hamman-Rich syndrome" OR bagassosis OR histiocytosis OR "fibrotic interstitial lung disease" OR "fibrotic lung disease")

**#2** Search (“exercise” OR “walking” OR “aerobic training” OR “endurance training” OR “interval training” OR “high-intensity training” OR “resistance training” OR “strength training”)

**#3** Search (#1 AND #2)
